# Supplementary material for: Common miRNAs of Osteoporosis and Fibromyalgia: A Review
Source: Int J Mol Sci. 2023 Aug 31;24(17):13513. doi: 10.3390/ijms241713513 (PMC10488272; doi:10.3390/ijms241713513)
Supplement: Supplementary file 1 [file ijms-24-13513-s001.zip › ijms-2525777-supplementary.pdf]

Table S1: List of dysregulated miRNAs (n = 46) in studies comparing fibromyalgia patients to healthy volunteers (miRNAs are presented according to their nomenclature).

**Differentially expressed microRNAs in fibromyalgia**			
**microRNA**	**No. studies**	**Regulation**	**References**
Same direction for FM and OP			
miR-1	n = 1	↓	[28]
let-7a-5p	n = 1	↓	[29]
miR-9-5p	n = 1	↑	[30]
miR-29a-3p	n = 1	↓	[31]
miR-128-3p	n = 1	↑	[30]
miR-328-3p	n = 1	↓	[30]
miR-335-5p	n = 1	↑	[32]
Opposite direction for FM and OP			
miR-7-5p	n = 1	↑	[30]
miR-23b-3p	n = 1	↓	[31]
miR-107	n = 2	↓	[29,33]
miR-139-5p	n = 1	↓	[28]
miR-143-3p	n = 1	↓	[34]
miR-151a-5p	n = 1	↓	[29]
miR-186-5p	n = 1	↑	[30]
miR-338-3p	n = 1	↓	[34]
miR-766-3p	n = 1	↓	[30]
Same or opposite direction for FM and OP			
miR-21-5p	n = 1	↓	[31]
miR-23a-3p	n = 1	↓	[28]
miR-133a	n = 1	↓	[28]
miR-145-5p	n = 2	↓	[31,34]
miR-148a-3p	n = 1	↑	[30]
miR-320a	n = 1	↑	[29]
miR-532-3p	n = 1	↓	[30]
Other miRNAs found			
let-7d	n = 1	↓	[35]
miR-27b-3p	n = 1	↑	[30]
miR-30b-5p	n = 1	↓	[29]
miR-101-3p	n = 1	↑	[30]
miR-103a-(3p)	n = 3	↓	[29,33,35]
miR-125a-5p	n = 1	↑	[33]
miR-130a-3p	n = 1	↓	[33]
miR-142-3p	n = 1	↓	[29]

miR-146a	n = 1	↓	[35]
miR-182-5p	n = 1	↑	[30]
miR-183-5p	n = 1	↑	[30]
miR-223-3p	n = 2	↓	[31,34]
miR-320b	n = 1	↓	[28]
miR-346	n = 1	↓	[28]
miR-374a-5p	n = 1	↑	[30]
miR-374b-5p	n = 1	↓	[29]
miR-451a	n = 1	↓	[34]
miR-454-3p	n = 1	↑	[30]
miR-548d-5p	n = 1	↑	[30]
miR-625-5p	n = 1	↓	[30]
miR-671-5p	n = 1	↓	[30]
miR-1275	n = 1	↓	[30]
miR-3609	n = 1	↓	[30]

Table S2: List of dysregulated miRNAs (n = 166) in studies comparing osteoporosis patients to healthy volunteers (miRNAs are presented according to their nomenclature).

**microRNA**	**No. studies**	**Regulation**	**References**
Same direction for FM and OP			
miR-1-3p	n = 2	↓	[36,37]
let-7a-3p	n = 1	↓	[37]
miR-9-(3p or 5p)	n = 2	↑	[38,39]
miR-29a-3p	n = 1	↓	[23]
miR-128	n = 1	↑	[40]
miR-328-3p	n = 1	↓	[41]
miR-335-5p	n = 2	↑	[42,43]
Opposite direction for FM and OP			
miR-7-5p	n = 1	↓	[42]
miR-23b-(3p)	n = 2	↑	[44,45]

miR-107	n = 1	↑	[46]
miR-139-5p	n = 1	↑	[44]
miR-143	n = 1	↑	[47]
miR-151a-3p	n = 2	↑	[48,49]
miR-186-5p	n = 1	↓	[42]
miR-338-3p	n = 1	↑	[50]
miR-766-5p	n = 1	↑	[51]
Same or opposite direction for FM and OP			
miR-21-(5p)	n = 12	↑↓	[23,41,43,52–0]
miR-23a-(3p or 5p)	n = 8	↑↓	[23,41,43,52,53,56,59,61]
miR-133a	n = 5	↑↓	[37,57,62–64]
miR-145-(3p or 5p)	n = 2	↑↓	[65,66]
miR-148a-(3p)	n = 5	↑↓	[56,59,66–68]
miR-320a	n = 4	↑↓	[42,53,69,70]
miR-532-(3p or 5p)	n = 4	↑↓	[42,43,71,72]
Other miRNAs found			
let-7b-5p	n = 1	↓	[42]
let-7c	n = 1	↑	[73]
let-7g-5p	n = 2	↓	[41,74]
miR-10a-3p	n = 2	↑	[75,76]
miR-10b-5p	n = 3	↑↓	[37,41,66]
miR-15a-5p	n = 1	↓	[77]
miR-15b-5p	n = 1	↓	[66]
miR-16-5p	n = 1	↓	[42]
miR-17-5p	n = 2	↑	[37,44]
miR-17-92a	n = 1	↓	[78]
miR-18a-3p	n = 2	↑	[79,80]
miR-19a-3p	n = 2	↓	[42,81]
miR-19b-3p	n = 4	↑↓	[42,43,66,82]
miR-22-3p	n = 3	↓	[41,74,83]
miR-24-(3p)	n = 3	↑	[56,59,84]
miR-25-(3p or 5p)	n = 3	↑	[51,59,85]
miR-26a	n = 1	↑	[86]
miR-27a-(3p)	n = 4	↑↓	[87–90]
miR-29b-3p	n = 3	↓	[42,91,92]
miR-30(a-5p)	n = 3	↑↓	[93–95]
miR-30e-5p	n = 1	↓	[42]
miR-31	n = 1	↑	[96]
miR-34c-3p	n = 1	↑	[37]
miR-92b-3p	n = 1	↓	[66]

miR-93-(5p)	n = 4	↑↓	[42,56,59,97]
miR-96	n = 2	↑	[46,94]
miR-99b-5p	n = 1	↓	[75]
miR-100-(5p)	n = 7	↑	[37,41,56,59,84,98,99]
miR-106b-5p	n = 1	↓	[66]
miR-122(a-5p)	n = 5	↑↓	[56,58,59,67,100]
miR-124(a-3p)	n = 3	↑	[23,56,59]
miR-125b-(5p)	n = 9	↑↓	[41,52,56,58,59,84,94,101,102]
miR-127-(3p or 5p)	n = 3	↑↓	[38,41,103]
miR-133b	n = 1	↓	[37]
miR-135a-5p	n = 1	↑	[104]
miR-135b-5p	n = 1	↑	[105]
miR-137-(3p)	n = 2	↑	[38,106]
miR-138-5p	n = 1	↑	[38]
miR-140-(3p or 5p)	n = 3	↑↓	[42,44,107]
miR-144-(3p or 5p)	n = 4	↑↓	[66,84,108,109]
miR-150-(5p)	n = 2	↑↓	[110,111]
miR-152-3p	n = 2	↓	[42,43]
miR-155-(5p)	n = 3	↑↓	[112–114]
miR-181a-2-3p	n = 1	↑	[75]
miR-181c-5p	n = 1	↓	[115]
miR-187	n = 1	↓	[116]
miR-193b-(3p or 5p)	n = 1	↓	[37]
miR-194-5p	n = 1	↑	[117]
miR-195	n = 1	↓	[110]
miR-197-3p	n = 1	↑	[44]
miR-200a-3p	n = 1	↑	[118]
miR-200b-3p	n = 1	↑	[38]
miR-203	n = 2	↓	[119,120]
miR-205-5p	n = 2	↑	[121,122]
miR-206	n = 3	↑↓	[37,38,123]
miR-208a-3p	n = 1	↑	[112]
miR-210-3p	n = 1	↑	[124]
miR-211-5p	n = 1	↓	[125]
miR-214-(3p)	n = 2	↑↓	[37,126]
miR-215-5p	n = 2	↓	[42,127]
miR-217	n = 2	↑	[128,129]
miR-219a-2-3p	n = 1	↑	[38]
miR-221-(5p)	n = 2	↓	[130,131]
miR-222	n = 1	↑	[110]
miR-224-3p	n = 1	↑	[51]
miR-302a-3p	n = 1	↓	[51]
miR-324-3p	n = 1	↓	[42]

miR-340-5p	n = 1	↑	[109]
miR-361-5p	n = 1	↑	[132]
miR-363-3p	n = 1	↑	[133]
miR-365a-3p	n = 3	↑↓	[42,114,134]
miR-373	n = 1	↓	[135]
miR-374c-3p	n = 1	↓	[66]
miR-375	n = 2	↑	[43,136]
miR-378a-5p	n = 1	↓	[42]
miR-378g	n = 1	↓	[137]
miR-410	n = 1	↑	[138]
miR-422a	n = 1	↑	[139]
miR-423-5p	n = 1	↑	[114]
miR-455-3p	n = 1	↓	[140]
miR-483-(3p or 5p)	n = 4	↑↓	[69,141–143]
miR-485-5p	n = 1	↑	[144]
miR-486-3p	n = 1	↓	[145]
miR-495-3p	n = 1	↓	[38]
miR-497-5p	n = 3	↑↓	[114,115,146]
miR-498	n = 1	↑	[147]
miR-499a-5p	n = 1	↓	[37]
miR-500a-5p	n = 1	↓	[38]
miR-503	n = 1	↓	[148]
miR-506-3p	n = 1	↑	[109]
miR-518(f)	n = 2	↑↓	[41,116]
miR-548aa	n = 1	↑	[37]
miR-548t-3p	n = 1	↑	[37]
miR-548x-3p	n = 1	↑	[149]
miR-550a-3p	n = 1	↓	[42]
miR-579-3p	n = 1	↑	[150]
miR-582-3p	n = 1	↑	[151]
miR-627-5p	n = 1	↑	[37]
miR-629-5p	n = 1	↑	[37]
miR-637	n = 1	↑	[112]
miR-642a-3p	n = 1	↑	[51]
miR-655-3p	n = 1	↓	[75]
miR-668-3p	n = 1	↓	[75]
miR-708-(5p)	n = 2	↑↓	[152,153]
miR-758-3p	n = 1	↓	[75]
miR-760	n = 1	↑	[37]
miR-874-3p	n = 1	↓	[154]
miR-885-5p	n = 1	↑	[44]
miR-889-3p	n = 1	↑	[155]
miR-920	n = 1	↓	[156]

miR-1227-3p	n = 1	↑	[44]
miR-1249-5p	n = 1	↓	[157]
miR-1270	n = 1	↑	[158]
miR-1271-5p	n = 2	↑↓	[38,159]
miR-1286	n = 1	↑	[160]
miR-1297	n = 1	↑	[161]
miR-1299	n = 1	↓	[75]
miR-1915-5p	n = 1	↓	[38]
miR-2278	n = 1	↓	[75]
miR-2861	n = 3	↑↓	[23,41,162]
miR-3065-5p	n = 1	↑	[50]
miR-3119	n = 1	↓	[38]
miR-3168	n = 1	↓	[38]
miR-3190-5p	n = 1	↓	[75]
miR-3191-3p	n = 1	↓	[75]
miR-3656	n = 1	↑	[37]
miR-3678-3p	n = 1	↓	[75]
miR-3679	n = 1	↑	[163]
miR-3690	n = 1	↓	[75]
miR-4274	n = 1	↑	[163]
miR-4516	n = 1	↓	[100]
miR-4665-3p	n = 1	↑	[94]
miR-4731-3p	n = 1	↓	[38]
miR-4767	n = 1	↓	[38]
miR-5009-5p	n = 1	↑	[75]
miR-5914	n = 1	↑	[94]
miR-6511a-3p	n = 1	↑	[37]
miR-6741-3p	n = 1	↑	[38]
miR-4746-3p	n = 1	↓	[38]
miR-6803-5p	n = 1	↑	[38]
miR-6851-3p	n = 1	↑	[109]
miR-6886-3p	n = 1	↓	[38]
miR-8068	n = 1	↑	[109]

Table S3: Characteristics of fibromyalgia studies with miRNAs in common with osteoporosis studies (n = 7)

First author, year	Tissue	Extraction Detection	Study design	Sample size	Age (years)	Gender	Primary outcome	Reference
Bjersing et al., 2013	cerebro spinal fluid	Q<br>q	case-control	10 FM<br>8 HV	48.5<br>58.5	FM: 100% F<br>HV: NK	To identify cerebrospinal miRNAs with expression specific for FM and to determine their correlation to pain and fatigue.	[31]
Bjersing et al., 2015	serum	Q<br>q	case-control	20 FM<br>20 HV	52.5	100% F	To identify characteristic miRNAs in FM and relations of specific miRNAs with characteristic symptoms	[29]
Braun et al., 2020	white blood cell	Q<br>q	case-control	31 FM<br>16 HV	50.7<br>NK	FM: 100% F<br>HV: NK	To identify miRNA signatures distinguishing patient clusters with FM.	[33]
Cerdá-Olmedo et al., 2015	peripheral blood monocellular cells	ITF<br>q	case-control	11 FM<br>10 HV	50.7<br>47.5	FM: 100% F<br>HV: NK	To identify changes in miRNA expression profiles (miRNome) of FM patients for the development of a quantitative diagnostic method of FM	[34]
Clos-Garcia et al., 2019	serum	NK<br>m	case-control	36 FM<br>36 HV	52,52 ± 10,3<br>53,5 ± 12,4	FM: 69,5% F, 30,48% M<br>HV: 48,15% F, 51,85% M	To identify potential molecular biomarkers for FM diagnosis and characterization, with different omics technologies.	[32]
Erbacher et al., 2022	whole blood	TF<br>q	case-control	49 FM<br>25 HV	53	100% F	To profile microRNA regulators of cholinergic transcripts (CholinomiRs)	[30]
Masotti et al., 2017	serum (& saliva)	Q<br>q	case-control	14 FM<br>14 HV	NK	100% F	To investigate the c-miRNA profiles in FM, correlating their expression with clinical and clinimetric parameters and to suggest a mathematical model for the diagnosis of FM	[28]

Abbreviations: c-miRNA, circulating miRNA; F, Female; FM, fibromyalgia; HV, Healthy Volunteers; ITF, Invitrogen Thermo Fisher; M, Male; m, multiplex assays; NK, not know; q, Quantitative Reverse Transcription Polymerase Chain Reaction; Q, Qiagen; TF, Thermo Fisher

Table S4: Characteristics of osteoporosis studies with miRNAs in common with fibromyalgia studies (n = 38)

First author, Year	Tissue	Extraction Detection	Study design	Gender	Sample size	Age (years)	Primary outcome	Reference
Al-Rawaf et al., 2021	serum	Q<br>q	cross-sectional	100% F<br>PM	55 OP<br>45 HV	63.9 ± 3.2<br>62.6 ± 4.1	Potential diagnostic role of circulating miRNAs, miR-148a and miR-122-5p, in the pathogenesis of OP and its association with bone markers, hypercortisolism, and vitamin D deficiency	[67]
Bedene et al., 2016	plasma	Q<br>q	case-control	100% F<br>PM	17 OP<br>57 HV	62<br>61	To identify c-miRNAs which could serve as potential biomarkers.	[68]
Chen et al., 2018	serum	ITF<br>q	cross-sectional	NK	9 OP<br>9 HV	69.2 ± 2.7<br>67.1 ± 2.6	To find the early diagnostic marker for OP using c-miRNAs	[41]
Chen et al., 2019	serum	Q<br>q	case-control	100% F<br>PM	46 OP<br>13 HV	69.6 ± 5.6<br>68.9 ± 6.5	To examine the difference in bone and muscle specific c-miRNAs in PM women based on their bone and muscle status, and to determine the associations between these specific c-miRNAs and muscle and bone variables	[52]
Ciuffi et al., 2022	serum	Q<br>NGS	case-control	OP: 74% F, 26% M<br>HV: 73.3% F, 36.7% M	50 OP<br>30 HV	68.0 ± 4.9<br>67.2 ± 5.0	Measure global miRNA expression in OP vs normal BMD.	[53]
Cong et al., 2020	plasma	ITF<br>q	cross-sectional	42 F, 24 M<br>for OP and HV	66 OP<br>66 HV	54,1 ± 5,3<br>54,3 ± 5,2	To investigate the interactions between miR-21 and GAS5	[54]
De-Ugarte et al., 2015	bone	Q<br>q	cross-sectional	100% F<br>PM	6 OP<br>6 HV	75,16 ± 3,54<br>72,5 ± 7,42	To identify miRNAs differentially expressed in fractured vs non-fractured bones.	[69]
Fu et al., 2020	whole blood	ITF<br>q	cross-sectional	100% F	11 PM OP<br>11 HV	NK	To investigate the expression level of miR-151a-3p and asses it effects on bone formation and resorption	[48]
Fu et al., 2021	serum	ITF<br>q	cross-sectional	OP: 73.2% F, 26.8% M<br>HV: 73.4% F, 26.6% M	82 OP<br>79 HV	50.48 ± 3.5<br>49.68 ± 4.17	To investigate the effects of ROR and miR-145-5p on the proliferation and apoptosis of osteoblasts.	[65]

Table S4: Continued

First author, Year	Tissue	Extraction Detection	Study design	Gender	Sample size	Age (years)	Primary outcome	Reference
Gao et al., 2018	hBMSCs	ITF<br>q	cross-sectional	2 F, 1 M for OP and HV	3 OP<br>3 HV	47 - 66<br>45 - 63	To investigate whether MALAT1 regulates Osterix expression by sponging miR-143 to promote hBMSCs osteogenic differentiation.	[47]
Gu et al., 2020	hBMSCs	ITF<br>s	cross-sectional	100% F	29 PM OP<br>20 HV	67.5 ± 9.52<br>65.27 ± 7.63	To identify miRNAs and investigate the role of miR-1-3p in the shift from OP and adipogenesis of hBMSCs in vitro and bone metabolism in vivo**.**	[36]
Guo et al., 2020	bone	ITF<br>q	cross-sectional	100% F<br>PM	10 OP<br>10 HV	62.15 ± 3.54<br>63.44 ± 6.07	To investigate whether miR-532-5p plays a role in the regulation of osteoporosis.	[71]
He et al., 2021	serum	ITF<br>q	cross-sectional	100% F<br>PM	15 OP<br>15 HV	57 - 80<br>61 - 76	To explore the biological functions of miRNA-151a-3p in OP	[49]
Jiang et al., 2020	hBMSCs	NK<br>q	cross-sectional	100% F<br>PM	25 OP<br>25 HV	≥ 65	To determine the role and mechanism of miR-23 with respect to regulating the osteogenic differentiation of hBMSCs.	[61]
Jiang et al., 2018	hBMSCs	ITF<br>q	cross-sectional	NK	NK	NK	To explore whether hBMSCs-derived exosomes extracted from OP patients could inhibit osteogenesis via microRNA-21/SMAD7.	[55]
Kelch et al., 2017	Serum & bone	Q<br>q	case-control	OP: 50% F, 50% M<br>HV: 50% F, 50% M	14 OP<br>14 HV	80<br>71.4	To determine the correlation of miRNAs with BMD and gender and study the intracellular expression of these miRNAs in different bone cell populations.	[56]
Kocijan et al., 2016	whole blood	Q<br>q	case-control	OP: 55.6% F, 4.4% M<br>HV: 59% F, 41% M	36 idiopathic OP & low-traumatic fractures<br>39 HV	46.6 ± 13.0<br>46.6 ± 9.4	Evaluation of c-miRNA signatures with idiopathic and PMOP low-traumatic fractures	[42]
Kong et al., 2021	plasma	Q<br>s	case-control	100% F<br>PM	5 OP<br>5 HV	57,6 ± 2,88<br>55,20 ± 3,27	To explore the role of plasma miRNAs in exosomes in early PM women with various bone mineral densities	[51]

Table S4: Continued

First author, Year	Tissue	Extraction Detection	Study design	Gender	Sample size	Age (years)	Primary outcome	Reference
Li et al., 2014	plasma	Q<br>q	case-control	100% F<br>PM	40 OP<br>40 HV	57.5 ± 11.3<br>56.5 ± 10.5	To examine the levels of miR-21, miR-133a and miR-146a in the plasma of PM women and investigate with sensitivity to BMD whether these plasma miRNAs levels correlate	[57]
Li et al., 2018	whole blood	Q<br>q	cross-sectional	100% F<br>PM	10 OP<br>10 HV	59 - 80<br>62 - 75	To assess miR-133 expression in serum isolated from PMOP patients	[62]
Li et al., 2021	serum	ITF<br>q	cross-sectional	NK	10 PM OP<br>10 HV	NK	To explore the function of lncRNA H19 in estrogen-induced osteogenic differentiation of hBMSCs	[72]
Lin et al., 2019	serum	Q<br>q	cross-sectional	100% F<br>PM	15 OP<br>15 HV	58 - 65<br>58 - 65	To identify circulating miR-338 associated with PMOP and perform functional validation *in vivo* and *in vitro.*	[50]
Liu et al., 2018	serum	ITF<br>q	cross-sectional	NK	5 OP<br>5 HV	NK	To research whether miR-96 can regulate osteogenic differentiation of BMSCs and be regarded as a biomarker in serum for OP	[46]
Lv et al., 2015	hBMSCs	ITF<br>q	cross-sectional	100% F	5 PM OP<br>5 HV	51 – 59<br>41 - 46	To explore how estrogen deficiency affects miR-133 expression and how miR-133 is involved in osteogenic differentiation of hBMSCs	[63]
Panach et al., 2015	serum	Q<br>q	case-control	100% F	8 OP<br>5 HV	79.6 ± 3.1<br>63.4 ± 8.1	To identify specific miRNAs in OP bone fracture patients compared to those in osteoarthritic controls	[58]
Ramírez-Salazar et al., 2018	serum	Q<br>q	case-control	100% F<br>PM	20 OP<br>20 HV	73.75 ± 4.46<br>71.1 ± 3.72	To analyze the expression of miRNAs in serum of patients with OP and HV.	[44]
Seeliger et al., 2014	serum & bone	Q<br>q	case-control	OP: 70% F, 30% M<br>HV: 100% F	10 OP<br>10 HV	79.3<br>78.8	To identify specific miRNAs in patients with OP fractures compared with non-OP fractures	[59]
Shao et al., 2020	serum exosome	ITF<br>s	case-control	100%<br>PM	12 OP<br>6 HV	NK	To evaluate the role of exosomal miRNAs in the diagnosis and prediction of OP in PM females with OP.	[38]

Table S4: Continued

First author, Year	Tissue	Extraction Detection	Study design	Gender	Sample size	Age (years)	Primary outcome	Reference
Suarjana et al., 2019	serum	ATF<br>q	cross-sectional	100% F	60 PM OP<br>60 HV	62<br>58.5	To determine the role of serum miR-21 expression, concentration of RANKL, OPG, TGF-β1, sclerostin and serum calcium, RANKL/OPG ratio, and physical activity on BMD of spine in hypoestrogenic PMOP compared with no osteoporosis	[60]
Sun et al., 2020	plasma	ITF<br>q	cross-sectional	OP: 91.7% F, 8.3% M<br>HV: 66.7% F, 33.3% M	12 OP<br>4 HV	68.9<br>47.8	To investigate miRNAs in the plasma of OP patients ± VF compared with non-osteoporotic HV subjects and to discover a role for miR-19b in osteogenesis	[66]
Teng et al., 2021	plasma	ITF<br>q	cross-sectional	100% F<br>PM	28 OP<br>25 HV	60.8 ± 6.86<br>50.1 ± 3.65	To analyze the involvement of TUG1 in OP and to explore the interaction between TUG1 and miR-23b in the osteogenic differentiation of human hBMSCs.	[45]
Wang et al., 2012	circulating monocytes	ATF<br>q	case-control	100% F<br>PM	10 OP<br>10 HV	61.6 ± 2.6<br>63.6 ± 3.2	To find significant miRNA biomarkers in human circulating monocytes underlying PMOP.	[64]
Wang et al., 2020	hBMSCs	ITF<br>q	cross-sectional	100% F	30 PM PO<br>20 HV	NK	To determine how lncRNA DANCR, miR-320a, and CTNNB1 interact with each other and regulate osteogenic differentiation in OP.	[70]
Wang et al., 2020	whole blood	ITF, Q<br>s	cross-sectional	100% F	3 PM OP<br>3 HV	75,16 ± 3,54<br>72,5 ± 7,42	To find the potential differentially expressed miRNAs in PMOP	[37]
Yavropoulou et al., 2017	serum	Q<br>q	case control	100% F<br>PM	70 LBM ± VF<br>30 normal BMD	69.5 ± 7.0<br>68 ± 5	To investigate serum levels of specific miRNAs, know regulators of bone metabolism, in PM women with LBM ± VF.	[23]
Zarecki et al., 2020	serum	Q<br>q	case-control	100% F<br>PM	76 low BMD ± VF ± treatment<br>40 HV	69.03<br>68.8	To compare the levels of c-miRNAs in older women with OP VF, ± low BMD and HV, and to correlate miRNAs expression levels with bone turnover markers.	[43]

Table S4: Continued

First author, Year	Tissue	Extraction Detection	Study design	Gender	Sample size	Age (years)	Primary outcome	Reference
Zhang et al., 2019	whole blood	ITF<br>q	cross-sectional	NK	30 OP<br>30 HV	NK	To explore the role of miRNA-9-5p in regulating OP development and its underlying mechanism.	[39]
Zhao et al., 2019	bone	ITF<br>q	cross-sectional	100% F	40 PM OP with fractures<br>40 HV with fractures	NK	To explore the potential mechanism of miR-128 in OP	[40]

Abbreviations: ATF, Ambion Thermo Fisher; BMD, Bone Mineral Density; c-miRNAs, circulating miRNAs; CTNNB1, Catenin beta-1's gene; DANCR, Differentiation Antagonizing Non-Protein Coding RNA; F, female; FM, fibromyalgia; GAS5, Growth Arrest Specific 5; hBMSCs, Human Bone Marrow Mesenchymal Stem Cell; HV, Healthy volunteers; ITF, Invitrogen Thermo Fisher; LBM, low bone mass; MALAT1, metastasis associated lung adenocarcinoma transcript 1; M, male; NGS, next-generation sequencing; NK, not know; OP, osteoporosis; OPG, Osteoprotegerin; PM, post menopause; q, Quantitative Reverse Transcription Polymerase Chain Reaction; Q, Qiagen; RANKL, receptor activator of nuclear factor- κB ligand; ROR, regulator of reprogramming; s, sequencing ; TGF-β1, Transforming growth factor beta 1; TUG1, taurine up-regulated 1; VF, vertebral fracture
